# Fungi between threat and promise: global perspectives on health and innovation

**DOI:** 10.3389/fmicb.2026.1743670

**Published:** 2026-02-18

**Authors:** Hao Li, Yu-Yen Yang, Santosh Chokkakula, Kuppusamy Sathishkumar, Mohammed Mujahid Alam, Abdullah G. Al-Sehemi, Xiaoxi Zhang, Siomui Chong, Gnanaprakash Jeyaraj

**Affiliations:** 1 Graduate School, Guangzhou University of Chinese Medicine, Guangzhou, China; 2 Dr. Pong Dermatologic and Aesthetic Clinic, Taipei, Taiwan, China; 3 Department of Microbiology, Chungbuk National University College of Medicine and Medical Research Institute, Cheongju, Chungbuk, Republic of Korea; 4 Department of Biotechnology, Rathinam College of Arts and Science, Coimbatore, Tamil Nadu, India; 5 Department of Chemistry, College of Science, King Khalid University, Abha, Saudi Arabia; 6 Research Center for Advanced Materials Science, King Khalid University, Abha, Saudi Arabia; 7 Department of Dermatology, The First Affiliated Hospital of Jinan University and Jinan University Institute of Dermatology, Guangzhou, China; 8 Department of Dermatology, The University of Hong Kong-Shenzhen Hospital, Shenzhen, China; 9 Saveetha Medical College and Hospital, Saveetha Institute of Medical and Technical Sciences, Kancheepuram, Tamil Nadu, India

**Keywords:** antifungal resistance, biotechnology, climate resilience, fungal pathogenicity, One Health

## Abstract

Fungi play a dual role as indispensable ecological engineers and as major agents of disease in humans, animals, and plants. Recent estimates highlight their substantial impact, with millions of invasive infections annually and severe agricultural losses threatening food security. At the same time, fungi underpin ecosystem services such as decomposition, soil aggregation, and carbon sequestration, while also serving as prolific sources of enzymes, metabolites, and sustainable biomaterials. Advances in single-cell and spatial omics, cryo-electron microscopy, AlphaFold-based structural predictions, and machine learning applied to biosynthetic gene clusters are transforming the study of fungal pathogenicity, symbiosis, and metabolism. These approaches are shifting fungal research from descriptive biology toward predictive, translational pipelines that connect mechanistic insights to drug discovery, resistance management, and biotechnological innovation. Nevertheless, challenges remain, including antifungal resistance, climate-driven emergence of new pathogens, limited therapeutic options, and bottlenecks in scaling fungal applications for sustainability. Addressing these requires integrated One Health strategies that bridge clinical, agricultural, and environmental perspectives. By uniting structural biology, omics, genome editing, and computational tools within a global framework, fungal biology can be harnessed not only to mitigate emerging risks but also to drive innovations in medicine, agriculture, and green technologies.

## 1 Introduction

Fungi are fundamental components of Earth’s biosphere, acting as decomposers, mutualists, pathogens, and prolific producers of bioactive molecules. They underpin key ecosystem services such as nutrient cycling, soil formation, and organic matter turnover, while simultaneously exerting major impacts on food systems, biodiversity, and human and animal health. Despite their importance, the global burden of fungal disease has long been underestimated. Recent estimates indicate approximately 6.5 million invasive fungal infections annually, associated with nearly 3.8 million deaths worldwide, placing fungal diseases among the most serious yet underrecognized global health challenges (Denning, 2024).

Climate change and environmental disruption are reshaping fungal ecology, geographic distributions, and disease risk. Rising temperatures, extreme weather events, and habitat disturbance are increasingly associated with the emergence of thermotolerant and stress-adapted fungal lineages capable of infecting new hosts and environments (Seidel et al., 2024). These trends have prompted coordinated global responses, including the World Health Organization’s Fungal Priority Pathogens List, which ranks clinically relevant fungi to guide surveillance and research efforts. Antifungal resistance has emerged as a critical concern, now framed within a One Health perspective that links clinical, agricultural, and environmental drivers (Li et al., 2025). Importantly, fungal antimicrobial resistance (fAMR) is now increasingly framed within a One Health context, linking clinical, agricultural, and environmental drivers (Fisher et al., 2024).

Fungal pathogens affect a broad range of hosts beyond humans. Dermatophytes cause widespread infections in domestic animals, while dimorphic and opportunistic fungi infect wildlife and immunocompromised hosts. In some cases, fungal diseases exert ecosystem-level impacts, exemplified by chytridiomycosis in amphibians, which has contributed to global population declines and biodiversity loss (Brown and Ballou, 2024). Aquatic systems are similarly affected, with fungal and fungal-like pathogens causing disease in freshwater and marine organisms, including economically important aquaculture species (Das et al., 2025; Sarkar et al., 2022).

Beyond pathogenesis, fungi are keystone ecological players across terrestrial and aquatic environments. Advances in omics technologies have improved understanding of fungal roles in symbioses, decomposition, and nutrient cycling. While mycorrhizal associations are among the most studied fungal mutualisms, fungal symbioses extend to endophytes, lichens, insect–fungus partnerships, and animal-associated mycobiomes, illustrating the ecological versatility of fungi (Serrano et al., 2024). In freshwater and marine ecosystems, fungi regulate organic matter turnover and food-web dynamics, contributing to ecosystem stability and biogeochemical cycling (Cunliffe, 2023; Grossart et al., 2019). In terrestrial environments, fungal symbioses extend well beyond mycorrhizae to include endophytes, lichen-forming mutualisms, insect–fungus associations, and animal-associated mycobiomes, collectively illustrating the remarkable ecological breadth of fungal life (Liu H. Y. et al., 2025; van der Heijden et al., 2015).

At the same time, fungi offer substantial opportunities for sustainable innovation. Fungal biomass and mycoproteins are increasingly explored as alternative protein sources within circular bioeconomy frameworks, while fungi play central roles in food fermentation, agriculture, and biotechnology. Beneficial fungi function as biological control agents, biofertilizers, and biostimulants, supporting more sustainable primary production with reduced chemical inputs (Dalbanjan et al., 2024; George and Ray, 2023; Srivastava et al., 2023; Vega, 2018). Fungal enzymes and metabolites further contribute to industrial processes, environmental remediation, and pharmaceutical development.

Recent technological advances are accelerating progress across fungal biology. Deep learning–based structural prediction platforms, such as AlphaFold 3, extend structure-based analysis beyond experimentally resolved systems (Abramson et al., 2024a), while machine-learning approaches increasingly support genome mining and metabolite prioritization (Riedling et al., 2024). Together, these tools are transforming fungal research from descriptive studies toward predictive and translational frameworks. Complementing these approaches, enzyme-guided bioprocessing and fungal biodegradation strategies are being explored for plastic waste remediation and sustainable biomanufacturing, although life-cycle analyses continue to identify challenges related to enzyme stability, scalability, and process costs (Ibrahim et al., 2024).

**FIGURE 1:**
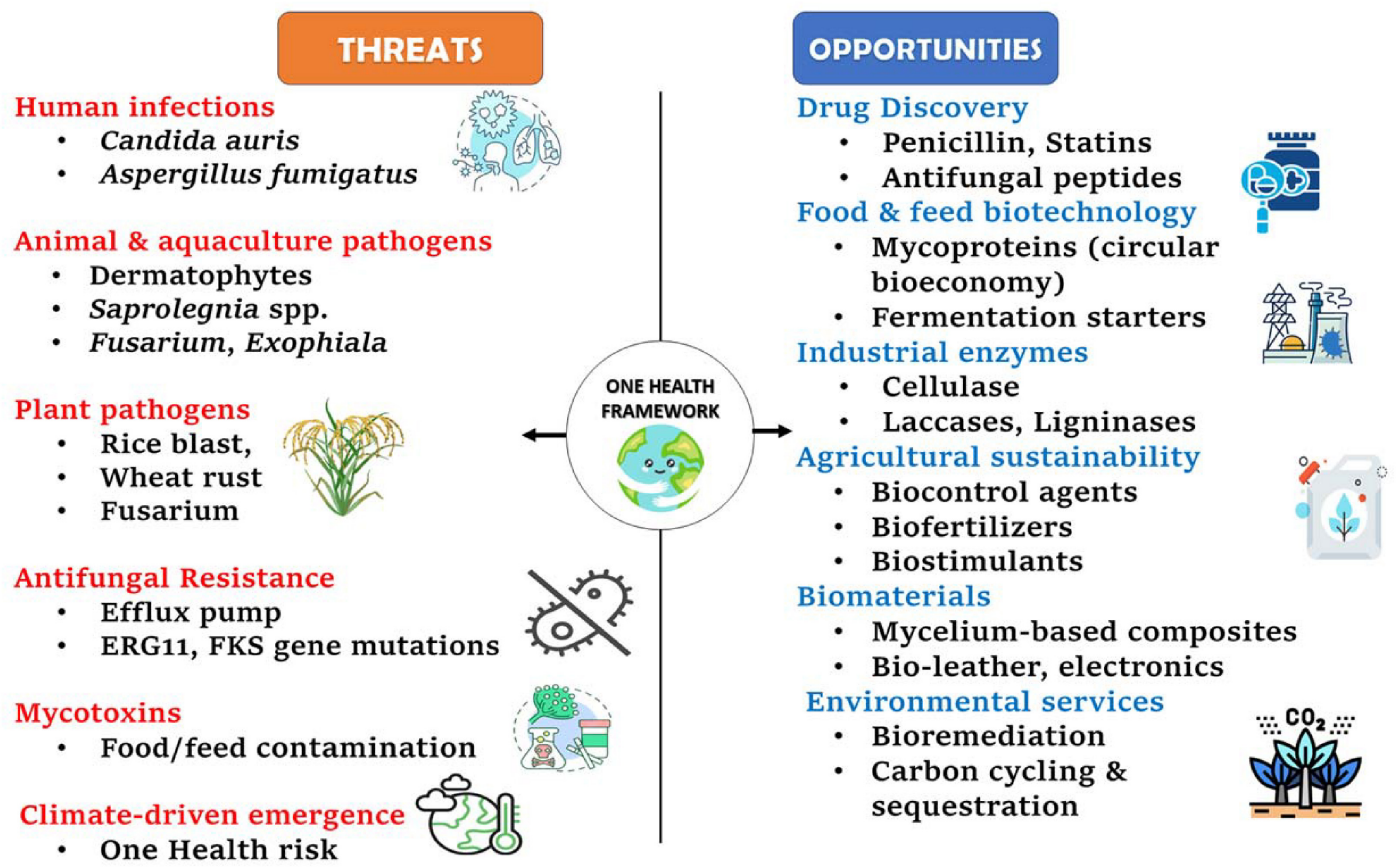
Fungi within a One Health framework, illustrating major threats and opportunities across human, animal, plant, environmental, and industrial domains. Threats include infectious diseases, antifungal resistance, mycotoxins, and climate-driven emergence, while opportunities span drug discovery, food and feed biotechnology, sustainable agriculture, industrial enzymes, biomaterials, and ecosystem services. Centre for Disease Prevention, E. (2025). *Survey on the epidemiological situation, laboratory capacity and preparedness for Candidozyma (Candida) auris, 2024*. https://doi.org/10.2900/2025052.

In this review, we synthesize recent advances in fungal biology across three interconnected domains: (i) pathogenicity, encompassing fungal diseases and antifungal resistance across human, animal, and plant hosts; (ii) ecology, including symbioses and ecosystem processes in terrestrial and aquatic environments; and (iii) biotechnology, highlighting fungal contributions to sustainable agriculture, industrial enzymes, and bio-based innovation. By integrating these perspectives within a One Health and sustainability framework, we aim to provide a balanced overview of fungi as both emerging global threats and powerful drivers of innovation. Figure 1 summarizes the dual roles of fungi as sources of emerging threats and as platforms for innovation across food systems, agriculture, health, and environmental sustainability.

## 2 Fungal pathogenicity: mechanisms and host interactions

### 2.1 Overview of fungal pathogenicity

Fungal pathogenicity involves a complex series of mechanisms that allow fungi to survive and thrive in various host environments, exploiting host vulnerabilities and adapting to different ecological niches. One of the defining features of many opportunistic fungi is their ability to form biofilms, which provide both physical barriers and metabolic hubs that contribute to immune evasion and resistance to antifungal treatments. Biofilm formation in pathogens such as *Candida auris* is particularly notable for its resilience in healthcare environments, where dry surface biofilms exhibit resistance to desiccation and hypochlorite tolerance, supported by upregulated transporters and iron acquisition pathways (Ware et al., 2025). Biofilms in fungi such as *C. auris*, along with other WHO-priority fungi, are known to exhibit broad-spectrum resistance to azoles, echinocandins, and disinfectants, making eradication efforts particularly challenging (Dire et al., 2023; Omardien and Teska, 2024; Pruitt et al., 2025).

Another cornerstone of fungal virulence is morphogenesis. Fungal pathogens often undergo dimorphic transitions, such as the yeast-to-hyphal switch seen in *Candida albicans* and *Histoplasma capsulatum*. These transitions are tightly regulated by conserved signaling pathways, including cAMP/PKA, MAPK/HOG, and calcium–calcineurin circuits, which enable fungi to adapt to the host environment (Ramírez-Sotelo et al., 2025). Additionally, environmental factors such as temperature and nutrient availability play significant roles in driving these transitions. Recent studies have shown how carbon metabolism and kinase regulation are integrated with morphogenetic programs, enhancing fungal invasion and tissue colonization (Martin-Vicente et al., 2024).

### 2.2 Human fungal pathobiology

Human fungal infections are significant contributors to morbidity and mortality, particularly in immunocompromised individuals. Pathogenic fungi such as *Candida*, *Aspergillus*, and *Cryptococcus* have evolved a range of virulence strategies to invade and persist in human hosts. One of the most critical features of human fungal pathogens is their ability to form biofilms *in vivo*, particularly in healthcare settings, where biofilm-associated infections are difficult to treat and contribute to chronic disease. The immune evasion strategies employed by these fungi include the secretion of extracellular vesicles, capsule formation, and melanin deposition, all of which help fungi evade immune detection and survive in hostile environments (Brown and Ballou, 2024; Rodrigues et al., 2025).

*Candida auris*, a multidrug-resistant pathogen, exemplifies the challenges posed by human fungal infections in clinical settings. Biofilm formation is central to its resilience, and recent studies show that its biofilms are highly resistant to antifungal treatments and disinfectants (Brown and Ballou, 2024). Moreover, its ability to survive on dry surfaces and in harsh conditions makes *C. auris* a persistent problem in hospitals and healthcare facilities (Ware et al., 2025). Dimorphic switching, such as the yeast-to-hyphal transition in *Candida albicans*, allows the pathogen to invade tissues and modulate the host immune system. The regulation of this transition involves key signaling pathways, such as cAMP/PKA, and environmental factors such as thermotolerance (Seidel et al., 2024; Ramírez-Sotelo et al., 2025).

Fungal pathogens also exploit host oxidative stress responses to survive in human tissues. In *Candida albicans*, the transcription factor Cap1 plays a crucial role in protecting the fungus from reactive oxygen species (ROS) generated by host immune cells (Swenson et al., 2024). Additionally, efflux pumps and mutations in antifungal drug targets, such as ERG11 in *C. auris*, contribute to resistance and persistence in human infections, complicating treatment regimens (Gandra et al., 2023; Pruitt et al., 2025).

### 2.3 Plant fungal pathobiology

In plants, fungal pathogens employ specialized strategies to invade host tissues, overcome plant immunity, and extract nutrients. Plant-pathogenic fungi like *Magnaporthe oryzae* (rice blast) and *Fusarium* spp. (wheat rust) are responsible for significant agricultural losses. A key feature of these pathogens is their ability to deliver effector proteins into plant cells, which manipulate host immune responses and promote fungal colonization. Effectors such as Pwl2 and MoCHT1 in *M. oryzae* have been shown to alter plasmodesmatal trafficking and chloroplast signaling, allowing the fungus to suppress plant defenses and facilitate infection (Liu C. et al., 2025; Were et al., 2025). These effector proteins are central to the concept of effector-triggered susceptibility, where the pathogen’s success relies on overcoming plant immune systems.

Furthermore, the immune response in plants, which involves pattern recognition receptors (PRRs), is often bypassed by the secretion of fungal effectors. These effectors can degrade plant defense proteins, alter hormone signaling pathways, and reprogram host cell metabolism to favor fungal growth (Oliveira-Garcia et al., 2023). Recent advancements in molecular biology have revealed that these effector molecules are critical for the co-evolution between plant and fungal species, with both developing and adapting countermeasures to the other’s strategies.

### 2.4 Fungal pathobiology in other hosts

Fungal pathobiology extends far beyond human and plant systems, encompassing complex and evolutionarily conserved infection strategies in insects, vertebrate wildlife, and aquatic hosts. These interactions reveal that fungal virulence is not host-specific but instead reflects flexible molecular programs that are redeployed across diverse biological contexts.

#### 2.4.1 Insect-associated fungal pathobiology

Entomopathogenic fungi such as *Beauveria bassiana* and *Metarhizium anisopliae* represent some of the most mechanistically well-characterized non-plant fungal pathogens. Infection begins with highly specific adhesion to the insect cuticle, mediated by hydrophobins and adhesins that recognize cuticular hydrocarbons, followed by localized secretion of cuticle-degrading enzymes, including subtilisin-like proteases (Pr1 family), chitinases, and lipases (Hong et al., 2024). Penetration is both enzymatic and mechanical, driven by turgor pressure generated within specialized infection structures analogous to plant appressoria.

Once inside the hemocoel, these fungi rapidly transition to yeast-like blastospores, a morphogenetic shift that enhances immune evasion and dissemination. At this stage, secondary metabolites such as destruxins, beauvericin, and bassianolide actively suppress insect cellular immunity by inhibiting hemocyte phagocytosis, melanization cascades, and antimicrobial peptide production (Lovett and St Leger, 2017). Transcriptomic and functional genomic studies reveal that many of these virulence determinants are conditionally expressed in response to host-derived cues, underscoring tight coupling between fungal metabolism, morphogenesis, and immune suppression. These same mechanisms underpin the successful deployment of entomopathogenic fungi as biological control agents, illustrating how virulence pathways can be repurposed for ecosystem-level interventions.

#### 2.4.2 Wildlife fungal pathogens and vertebrate hosts

Fungal diseases in vertebrate wildlife highlight how host physiology and environmental stressors intersect to shape disease outcomes. The chytrid fungi *Batrachochytrium dendrobatidis* and *Batrachochytrium salamandrivorans* exemplify a mode of pathobiology centered on epithelial disruption rather than invasive tissue colonization. These fungi infect keratinized layers of amphibian skin, where they interfere with ion transport processes essential for osmotic balance and cardiac function, ultimately causing mortality without deep tissue invasion (Fisher and Garner, 2020).

At the molecular level, chytrid pathogenesis involves secreted proteases that degrade host keratin and immune effectors, as well as modulation of host antimicrobial peptide responses. Disease severity is strongly influenced by environmental temperature and host microbiome composition, revealing that fungal virulence in wildlife is often emergent rather than intrinsic. Comparable dynamics have been reported for fungal infections in reptiles and fishes, where stress-induced immunosuppression and environmental perturbations facilitate opportunistic fungal colonization (Rathinam et al., 2024). These systems underscore that fungal pathobiology in animals frequently operates at the interface of host barrier tissues, immune modulation, and environmental change.

#### 2.4.3 Freshwater fungal pathobiology and trophic interactions

In freshwater ecosystems, fungi exert pathogenic and regulatory roles that are mechanistically distinct from terrestrial host interactions. Chytrid fungi parasitizing phytoplankton infect host cells through rhizoid-mediated penetration, hijacking host carbon and nutrients for fungal reproduction. Importantly, chytrid infections restructure aquatic food webs via the “mycoloop,” whereby fungal zoospores serve as a high-quality nutritional resource for zooplankton, redirecting carbon flow from otherwise inedible algal biomass to higher trophic levels (Kagami et al., 2014).

From a mechanistic perspective, these interactions are driven by precise host recognition, chemotaxis toward algal exudates, and rapid transcriptional reprogramming that supports motility, encystment, and host invasion. Genomic analyses indicate that aquatic chytrids possess streamlined genomes enriched for carbohydrate-active enzymes and host-interaction modules, reflecting specialization for parasitic lifestyles in nutrient-dilute environments (Grossart et al., 2019). Thus, fungal pathogenicity in freshwater systems is inseparable from ecosystem-scale biogeochemical processes.

#### 2.4.4 Marine fungal pathobiology and host associations

Marine fungi represent an increasingly recognized but still underexplored component of oceanic microbial ecosystems. These fungi associate with diverse hosts, including macroalgae, corals, sponges, and fish, where they can act as saprotrophs, mutualists, or opportunistic pathogens depending on host condition and environmental stress. Recent metagenomic and single-cell studies have revealed that marine fungi actively participate in particle-associated microbial consortia, degrading complex polysaccharides and contributing to carbon export processes in the biological pump (Breyer and Baltar, 2023).

Pathogenic interactions in marine systems often emerge under climate-driven stress, such as warming, acidification, and hypoxia, which compromise host immunity and shift fungal behavior from commensalism toward pathogenicity. Mechanistically, marine fungal pathogenicity involves stress-responsive gene networks, secreted enzymes, and secondary metabolites that enable colonization of host surfaces and tissues (Cunliffe, 2023; Peng X. et al., 2024). These findings reinforce that fungal virulence is context-dependent and dynamically regulated by environmental cues.

Taken together, fungal pathobiology in insects, wildlife, and aquatic systems demonstrates that virulence is an emergent property of regulatory integration across adhesion, morphogenesis, metabolism, immune modulation, and environmental sensing. These systems reveal conserved strategies such as morphogenetic switching, secreted effectors, and stress-adaptive metabolism deployed in host-specific configurations. Incorporating these non-terrestrial and non-human host contexts therefore strengthens the manuscript’s conceptual framework and aligns fungal biology with One Health and ecosystem-level perspectives.

## 3 Fungal metabolomics and analytical tools

Recent advances in fungal metabolomics have significantly enhanced our ability to uncover the diverse array of secondary metabolites produced by fungi, bridging the gap between genome-based predictions and experimental chemical identification. Metabolomics, particularly when integrated with genomics, transcriptomics, and structural biology, provides a holistic understanding of fungal biology, host interactions, and ecological roles. The primary tool for metabolomics is liquid chromatography coupled with tandem mass spectrometry (LC–MS/MS), which allows for high-sensitivity, untargeted profiling of metabolites in fungal extracts. This method has become the cornerstone for large-scale fungal metabolomic studies, enabling the identification of bioactive compounds involved in virulence, biofilm formation, and drug resistance in human pathogens such as *Aspergillus* and *Candida* species (Gonzalez-Jimenez et al., 2023; Liu M. et al., 2024)

Alongside LC–MS/MS, molecular networking platforms such as GNPS (Global Natural Products Social Network) and open-source software for untargeted metabolomics and lipidomics data analysis, including MS-DIAL, are increasingly used to annotate and compare fungal metabolite profiles. These tools allow for the high-throughput analysis of complex fungal metabolomes, accelerating the discovery of novel metabolites, especially those that may not be captured by traditional biosynthetic gene cluster predictions (Abramson et al., 2024a; Riedling et al., 2024). Through molecular networking, researchers can link spectral data across different fungal strains, environments, or developmental stages, uncovering hidden chemical diversity and facilitating the dereplication of known metabolites.

Moreover, *in silico* annotation tools such as SIRIUS and MS-DIAL enhance the ability to predict molecular structures from MS/MS data, offering powerful computational support for metabolomic studies. These tools employ advanced algorithms to predict molecular formulas and substructures directly from raw data, allowing researchers to accurately annotate and hypothesize the structures of fungal metabolites without reliance on pre-existing spectral libraries (Lai et al., 2025; Zhu et al., 2025). Such tools are crucial for the accurate interpretation of untargeted datasets, particularly when studying poorly characterized or novel fungal metabolites.

Integrating these cutting-edge techniques with fungal genomics provides a more comprehensive view of fungal metabolism. For example, biosynthetic gene cluster (BGC) prediction can identify regions of the genome that may encode for secondary metabolites, while metabolomics can validate these predictions by directly identifying the chemical products of these pathways (Lai et al., 2025). This integrated approach enables the functional annotation of fungal genomes, linking genetic potential to chemical production and fostering the discovery of novel bioactive compounds. Fungal metabolomics, when coupled with transcriptomics, can also reveal how environmental factors, host interactions, and stress conditions influence metabolic pathways, leading to the production of metabolites with distinct ecological and medical relevance (Reveglia et al., 2025).

The use of metabolomics is rapidly expanding beyond academic research into practical applications in drug discovery, agriculture, and biotechnology. Fungal metabolites such as gliotoxin from *Aspergillus* and fumagillin from *Fusarium* have already shown promise as anti-cancer agents, immunosuppressive agents, and industrial enzymes, demonstrating the potential of fungal specialized metabolites in diverse biotechnological applications (Günther et al., 2024; Alves et al., 2025). Moreover, their role in ecological processes, such as their contribution to plant-fungal interactions and biodegradation, underscores the broad impact of fungal metabolites on both human health and environmental sustainability.

## 4 Ecological roles of fungi

Fungi are architects of terrestrial ecosystems, coupling decomposition, soil aggregation, nutrient exchange, and stress buffering into emergent functions that regulate productivity and carbon balance. At the soil–carbon interface, fungi contribute not only via fast saprotrophic turnover but also through the formation and stabilization of microbial necromass, a relatively persistent carbon pool now recognized as a major component of global soil organic carbon (SOC). A recent meta-analysis across croplands estimated that fungal necromass accounts for more than 25% of SOC, with climate, soil texture, and management identified as major drivers, while residue retention and reduced tillage were shown to enhance necromass accrual (Table 1; Liu D. et al., 2024). Necromass persistence is promoted by its sorption to mineral phases and by physical protection within aggregates, demonstrating how fungal residues contribute to long-term carbon sequestration. Additional trait-level studies show that melanized fungal necromass is particularly resistant to microbial decomposition, further extending its role in soil carbon stabilization (Maillard et al., 2023).

**TABLE 1:** Fungal biology across domains: advances, challenges, and opportunities.

Domain	Recent advances	Major challenges	Future opportunities	Key references
Human health	*Candida auris* biofilm resilience and disinfectant tolerance; Cryo-EM structures of efflux pumps; Novel antifungals under trial (fosmanogepix, olorofim)	Multidrug resistance; limited diagnostics; inequities in LMICs	AI-driven efflux pump inhibitors for antifungal resistance; nanotechnology-based antifungals; global resistance surveillance	(Abramson et al., 2024a; Bassetti et al., 2025; Bhargava et al., 2025; Casalini et al., 2024; Chowdhary et al., 2020; Dire et al., 2023; Du et al., 2025; Hodges et al., 2025; Ibe and Pohl, 2024; Kriegl et al., 2025; Li et al., 2025; Omardien and Teska, 2024; Ware et al., 2025)
Plant health	Effector structural studies in *Magnaporthe oryzae*; CRISPR-based resistance breeding; Effector trafficking mechanisms resolved	Rapid pathogen evolution; regulatory hurdles; crop diversity gaps	CRISPRa for durable crop resistance; effector-informed resistance breeding	(Chen et al., 2024; Kainat et al., 2025; Le Naour-Vernet et al., 2025; Li et al., 2024; Liu C. et al., 2025; McLaughlin et al., 2025; Oliveira-Garcia et al., 2023; Shen et al., 2024; Singer et al., 2024; Wang et al., 2023; Were et al., 2025)
Ecology	Single-cell/spatial omics of mycorrhizal symbiosis; Necromass contributions to soil carbon sequestration; Drought resilience mechanisms	Over-interpretation of CMNs; biodiversity loss; climate-driven shifts	Mechanistic soil models; conservation genomics; climate-adapted microbiomes	(Canarini et al., 2024; Hannula and Veen, 2025; Hopkins et al., 2024; Ji et al., 2024; Karst et al., 2023; Knight et al., 2024; Liu D. et al., 2024; Maillard et al., 2023; Pena et al., 2023; Seidel et al., 2024; Serrano et al., 2024; Son et al., 2024; Zhou et al., 2024)
Biotechnology	ML-guided prioritization of fungal BGCs; CRISPR workflows in filamentous fungi; Mycelium-based materials for electronics and textiles	Scaling bio-based plastic degradation; enzyme stability bottlenecks; process cost-efficiency	Sustainable bioprocessing; enzyme engineering for lignin valorization; hybrid chem-bio remediation	(Bergeson et al., 2024; Camilleri et al., 2025; Fu et al., 2025; Ibrahim et al., 2024; Jiju et al., 2025; Karnwal et al., 2024; Lai et al., 2025; Mazurkewich et al., 2025; Meng et al., 2025; Pruckner et al., 2025; Riedling et al., 2024; Ropero-Pérez et al., 2024; Saad et al., 2024; Shankar et al., 2024; Shen et al., 2024; Steindorff et al., 2024; Thathola et al., 2024)

Aggregate formation itself is strongly mycorrhiza-mediated. Arbuscular mycorrhizal (AM) hyphae act as living scaffolds, while glomalin-related soil proteins (GRSP) impart hydrophobicity and adhesive properties that stabilize aggregates, thereby shielding organic matter and slowing decomposition. Recent studies have clarified the chemical properties of GRSP including glycosylation and metal adsorption that underpin carbon sequestration and soil remediation benefits (Son et al., 2024). Field-scale and modeling work further show that AM hyphal networks and glomalin production underpin aggregate hierarchy and the energetic landscapes of soil structure (Ji et al., 2024). These mechanisms explain how fungal traits directly translate into aggregate-protected carbon pools and improved soil fertility.

In plant–fungus symbioses, next-generation cellular atlases are now providing unprecedented resolution of mutualistic programs. Using integrated single-nucleus and spatial transcriptomics (Serrano et al., 2024), mapped discrete stages of AM symbiosis in Medicago truncatula–Rhizophagus irregularis roots, identifying cell-type-specific host responses and fungal modules during colonization. These insights provide a mechanistic blueprint for how nutrient exchange, cell identity, and immunity are coordinated at the cellular level, offering resources for breeding stress-resilient crops. Other studies highlight how carbon–nitrogen stoichiometry in ectomycorrhizal fungi governs host allocation, linking fungal nutrient economics to forest carbon cycling (Pena et al., 2023).

At ecosystem scales, fungi also help buffer against climate extremes. Large cross-biome analyses reveal that soil microbiomes display consistent responses to droughts, floods, and heat events, with edaphic context and community history influencing resilience (Knight et al., 2024; Zhou et al., 2024). Fungi often maintain growth during drought, in contrast to bacteria, by investing in lipid storage compounds and sustaining function under low water potential (Canarini et al., 2024). Field studies show that drought legacies interact with wildfires to restructure microbial communities, often reducing fungal and bacterial richness with increasing disturbance severity, yet fungal dominance can preserve decomposition and nutrient cycling when water is scarce (Hopkins et al., 2024; Hannula and Veen, 2025). These physiological and ecological patterns help refine models of soil carbon feedbacks under climate change.

Finally, claims that common mycorrhizal networks (CMNs) broadly redistribute carbon or information among trees are being re-evaluated. Karst et al. (2023) highlighted that positive-result bias and overinterpretation have outpaced causal evidence, urging rigorous experimental designs such as isotopic tracing and spatial exclusion controls before generalizing CMN effects. This cautious view does not deny CMN existence but situates their ecosystem influence within a testable framework. Undersampled fungal biodiversity in tropical regions limits conservation and biotechnological potential, requiring global collaboration to protect these ecosystems (Knight et al., 2024). Conceptual updates also refine CMN definitions, distinguishing between conditions in which they act as “socialist” facilitators of cooperation and when they resemble “capitalist” systems of partner choice (Ullah et al., 2024; Rillig et al., 2025).

In sum, recent advances connect molecular and cellular maps of symbiosis to soil-scale mechanisms such as necromass stabilization and aggregate formation, while also clarifying fungal physiological responses to climate extremes. These insights provide a predictive framework for managing fungal ecology to enhance soil health, carbon storage, and ecosystem resilience in the Anthropocene.

## 5 Fungi in biotechnology and sustainability

Fungi are shifting from “workhorse enzyme producers” to versatile biofoundries that enable drug discovery, green chemistry, advanced materials, and remediation. Three forces drive this shift: (i) genome mining + machine learning that reveals cryptic biosynthetic gene clusters (BGCs) and prioritizes them for activity; (ii) genome editing and strain engineering that tune pathways and secretion; and (iii) process/biomaterials innovation that translates enzymes and mycelium into scalable products.

### 5.1 Discovery engines: genome mining and machine learning

Large-scale BGC resources and ML models are now targeting fungal secondary metabolism specifically. Recent studies shows that models trained on bacterial BGCs can be adapted to predict fungal metabolite bioactivity with surprisingly high accuracies, and that integrating fungal BGC databases with ML prioritization accelerates triage of cryptic clusters (Riedling et al., 2024; Lai et al., 2025; Zhu et al., 2025). These tools complement classic antiSMASH-style mining and are beginning to rank tailoring enzymes and scaffolds most likely to yield novel chemistry (Zhang et al., 2025). Together, they provide a practical route from genome to candidate compound, shortening the loop to validation.

### 5.2 Editing and chassis optimization: CRISPR in filamentous fungi

CRISPR/Cas systems have matured from proof-of-concept to routine editing for many filamentous species. Recent studies outline expression strategies (plasmid, RNP, and *in vivo* sgRNA expression), multiplexing, and donor design for precise pathway rewiring and secretion enhancement (Ropero-Pérez et al., 2024; Shen et al., 2024). Practical bottlenecks low editing frequencies in some strains are being addressed: in Penicillium digitatum, workflow tweaks lifted CRISPR/Cas9 disruption efficiency from ∼10 to ∼83%, illustrating how selection and screening design can be decisive (Ropero-Pérez et al., 2024). These advances make it far more feasible to activate silent BGCs, knock out competing sinks, and tune redox/cofactor balance for metabolite titers. It should be noted, however, that despite rapid technical advances, the environmental release of genome-edited fungi or plants remains constrained in many regions by regulatory frameworks that treat genome-edited organisms similarly to genetically modified organisms. These legislative limitations represent a significant bottleneck for field deployment of edited fungal strains in agriculture and environmental applications, emphasizing the need to align technological progress with evolving regulatory and societal frameworks.

### 5.3 Industrial enzymes for the bioeconomy: thermostability, lignin, and assay realism

Comparative genomics of thermophilic fungi points to streamlined genomes enriched for thermostable carbohydrate-active enzymes (CAZymes) useful for harsh bioreactor conditions (Steindorff et al., 2024). At the enzyme scale, structural work and biophysical analyses continue to explain thermotolerance in cellulases (e.g., GH6 cellobiohydrolases) and glycoside hydrolase families relevant to crystalline cellulose attack (Yamaguchi et al., 2024; Mazurkewich et al., 2025). For lignin valorization, 2024–2025 reviews and research highlight engineering of fungal laccases and peroxidases, ML-guided discovery of alkaline basidiomycete laccases, and updated activity assays that better reflect industrial matrices and emerging contaminants key for translating lab hits into process-ready catalysts (Martin-Vicente et al., 2024; Singh et al., 2024; Thathola et al., 2024; Fu et al., 2025; Jiju et al., 2025). Collectively, these show a maturing pipeline from enzyme prospecting → property prediction/engineering → fit-for-purpose assays.

In addition to plant-based feedstocks, fungal enzymes underpin circular bioeconomy strategies that valorize diverse waste streams, including agro-food residues and marine-derived byproducts such as chitin-rich shellfish waste. Fungal hydrolases and oxidoreductases enable the conversion of these substrates into value-added products, supporting sustainable industrial bioprocessing beyond terrestrial biomass. Increasing attention is also focused on fungal enzymes involved in polymer and plastic biotransformation; filamentous fungi have been shown to promote low-density polyethylene deterioration, implicating ligninolytic, cutinolytic, and related enzyme systems in plastic valorization pathways. Together, these advances position fungal enzymes as versatile tools for waste-to-resource pipelines spanning biomass conversion, marine byproduct utilization, and plastic degradation within circular industrial frameworks (Spina et al., 2021; Temporiti et al., 2022; Dhiman et al., 2024; Ekanayaka et al., 2025).

### 5.4 Mycelium materials: from lab sheets to performance engineering

A surge of 2024–2025 work is transforming mycelium-based materials (MBMs) from concept to engineered products. Mechanical performance (Young’s modulus, tensile strength) can be substantially improved by post-growth cross-linking, densification, and composite lay-ups (d’Errico et al., 2024). Comprehensive reviews document design rules for mycelium composites and “leather-like” skins, scaling methods, and application niches (Shankar et al., 2024; Camilleri et al., 2025), while device-oriented studies show mycelium skins functioning as flexible, sustainable substrates for electronics (Pruckner et al., 2025). The overarching theme is control of hyphal density, moisture, and polymer cross-links to move from cork-like foams toward structural, barrier, or electronic materials.

### 5.5 Remediation: plastics and metals promise with hard edges

**FIGURE 2:**
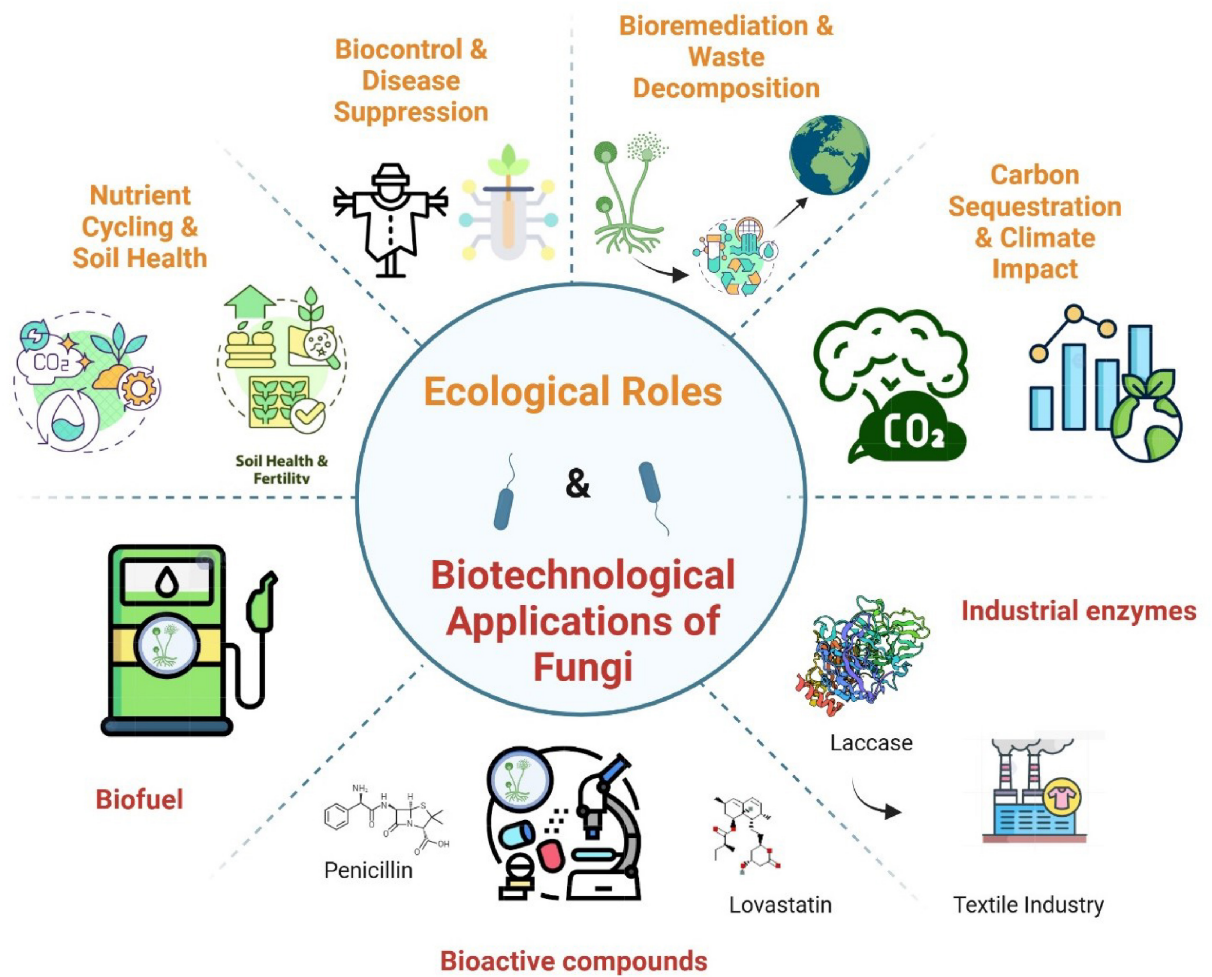
Ecological roles and biotechnological applications of fungi. This figure highlights the dual significance of fungi in ecosystems and biotechnology.

Fungal potential for plastic biodegradation has expanded through broad organismal screens that identify diverse ascomycetes and basidiomycetes with measurable activity against synthetic polymers (Ibrahim et al., 2024). Marine-adapted isolates further extend this capacity, with strains capable of initiating polyethylene surface erosion under controlled conditions (Vaksmaa et al., 2024). While such discoveries underscore the taxonomic breadth of fungal degraders, most reported activities remain modest, typically limited to superficial scarring or partial depolymerization. These limitations reflect intrinsic material barriers polymer crystallinity, hydrophobic backbones, and heterogeneous additive mixtures that impede enzymatic access. As emphasized in a critical perspective, overcoming these bottlenecks will likely require hybrid solutions that integrate enzyme engineering, co-culture systems, and chemical–biological workflows rather than relying on single-strain biocatalysis (Bergeson et al., 2024). Recent studies highlight how enzyme-guided strategies and mycological approaches can achieve degradation efficiencies in lab-scale setups but also caution that environmental variables strongly constrain reproducibility and scalability (Karnwal et al., 2024; Saad et al., 2024). Together, these studies situate fungal plastic degradation as a proof-of-concept domain: one that demonstrates genuine enzymatic versatility but must confront life-cycle assessments, process economics, and ecological safeguards before translation into industrial or environmental remediation contexts. Several ecological and biotechnological roles of fungi illustrated in Figure 2, including carbon sequestration, nutrient cycling and soil health, bioremediation and waste decomposition, biofuel production, and enzyme-driven industrial processes (e.g., textile applications), represent well-established or rapidly advancing areas of fungal research. While not all of these sectors are discussed in depth here, they collectively frame the broader functional landscape in which fungal biology operates and contextualize the more detailed discussions of pathogenicity, ecology, and biotechnology presented in this review.

## 6 Fungi and human health

Fungal pathogens continue to escalate not just in prevalence, but in their threat to public health systems globally. The WHO Fungal Priority Pathogens List (FPPL) has become more than a catalog it’s a roadmap for investment and policy (Casalini et al., 2024). However, recent analyses show that among the FPPL “critical” pathogens, *Candida auris* is not only expanding geographically, but outpacing surveillance and control capacity. The European Centre for Disease Prevention and Control (ECDC) reports that by 2023, over 4,000 *C. auris* infections or colonization cases were documented in EU/EEA countries, with some nations transitioning to regional endemicity in just 5–7 years after the first case (European Centre for Disease Prevention and Control, 2025). This underlines how rapidly *C. auris* can become entrenched.

Treatment options remain limited. A recent study, showcases that echinocandins are still first-choice for many *C. auris* infections, but high resistance to amphotericin and azoles persists, often leaving clinicians with few reliable agents (Kriegl et al., 2025). Case reports add nuance: for example, a ventriculitis case caused by *C. auris* was successfully treated using liposomal amphotericin B combined with flucytosine underscoring that older drugs, when appropriately combined and dosed, still have roles (Kang et al., 2024). But high toxicity and monitoring burdens make such regimens hard to scale.

Beyond absolute numbers, Simões et al. stress that fungal infections remain vastly underdiagnosed, with limited access to culture and molecular diagnostics in low- and middle-income countries obscuring the true burden. They identify candidiasis, aspergillosis, and cryptococcosis as major contributors to preventable mortality, highlighting that the global fungal disease burden is not only high but systematically underestimated (Simões et al., 2023). On the diagnostic and infection-control fronts, the Antifungal Resistance Review (Hui et al., 2024) emphasizes that delays in identification (misidentification, slow culture methods) contribute to worse outcomes. Many *C. auris* outbreaks in hospitals, especially ICUs, have been tied to colonization and environmental persistence fungus survives on surfaces and in hospital plumbing, resisting standard disinfection (Bassetti et al., 2025; Bhargava et al., 2025). ICUs managing critically ill COVID-19 patients reported spikes in *Candida auris* infections, largely driven by immune suppression, extensive use of broad-spectrum antibiotics and antifungals, prolonged mechanical ventilation, and lapses in infection-control practices (Chowdhary et al., 2020; Hoenigl et al., 2022; Najeeb et al., 2022).

Mechanistic work highlights that *C. auris’* resistance is often multifactorial: efflux pump overexpression, mutations in ERG11 (azole target), FKS gene mutations for echinocandin resistance, and biofilm matrix components all contribute (Bhargava et al., 2025). There is also growing concern that environmental azole exposure (e.g., in agriculture) is selecting resistance prior to clinical presentation. Azole resistance in *Aspergillus fumigatus*, driven by agricultural use, includes mutations like TR34/L98H, complicating treatment with high mortality (47–88%) (Simões et al., 2023).

Emerging therapeutic and preventative strategies are being explored: novel drugs are in trials (rezafungin, etc.), repurposed agents, combination therapies, and new disinfectant/formulation approaches. Du and collaborators (Du et al., 2025) describe innovative antifungal strategies, including natural antifungal compounds, antimicrobial peptides, and nanotechnology-based delivery systems. Meanwhile, Recent advances in antifungal research highlight the molecular classification of existing agents, emphasize limitations related to toxicity and spectrum gaps, and stress the urgent need for broad-spectrum compounds with favorable pharmacokinetics and minimal human toxicity (Li et al., 2025).

**FIGURE 3:**
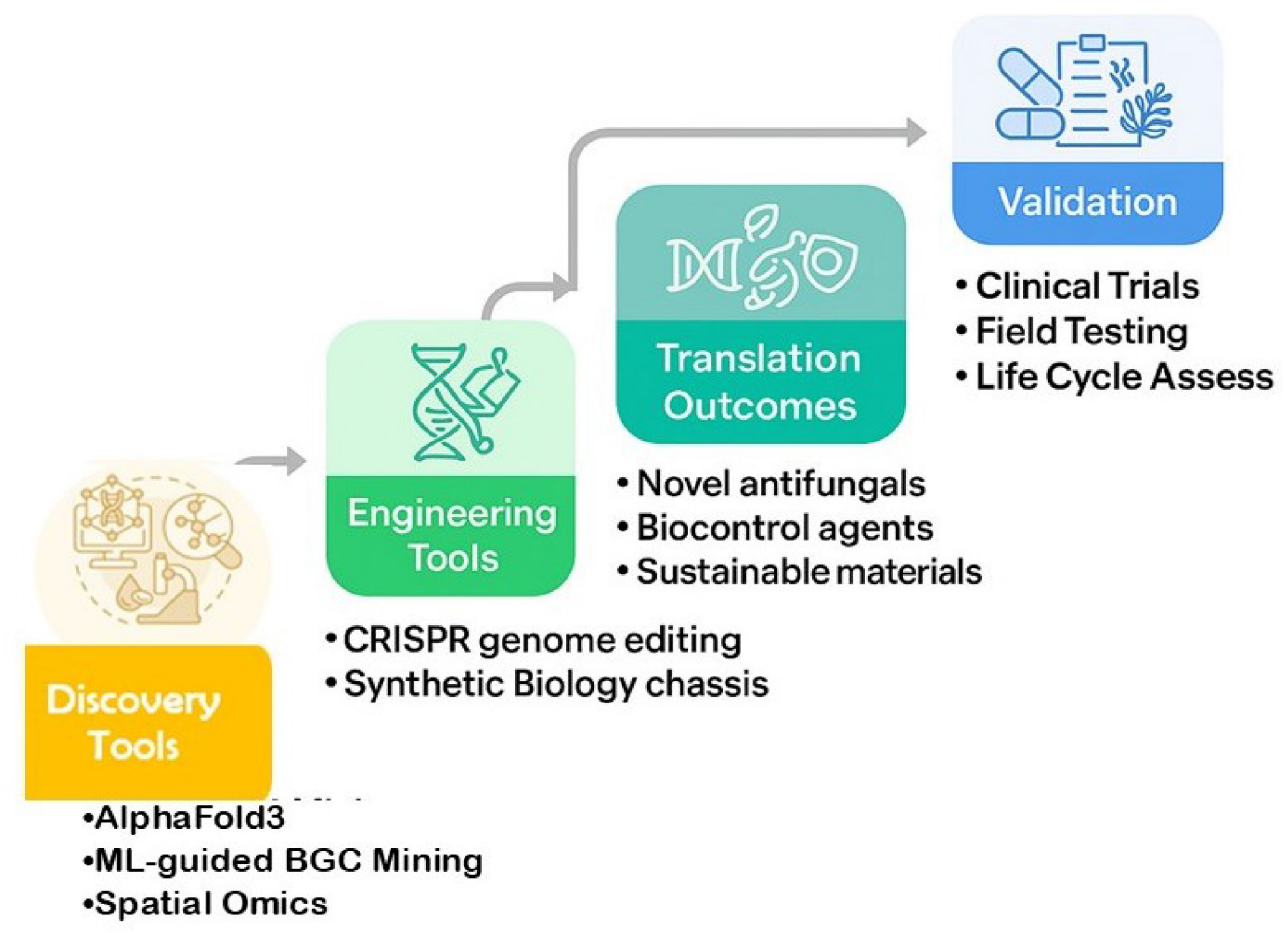
Technological pipeline for advancing fungal biology from discovery to translation.

Finally, public health challenges include underreporting, lack of standardized breakpoints (for some agents/pathogens), inequities in access to diagnostics and treatments in low- and middle-income countries, and environmental reservoirs contributing to spread. Limited access to advanced diagnostics in low-resource settings exacerbates fungal disease burdens, necessitating affordable, portable solutions to bridge these gaps (Bassetti et al., 2025). The rising case count, combined with limited options, High mortality, and rapid transmission, particularly in hospital environments, foreshadow potentially larger crises unless coordinated multidisciplinary responses ramp up (Figure 3; Casalini et al., 2024; Bassetti et al., 2025). Mycotoxins like aflatoxins and fumonisins, produced by *Aspergillus* and *Fusarium* species, exacerbate human health risks through contaminated food, causing carcinogenic and immunosuppressive effects, with global feed contamination rates reaching 70–97% for fumonisins in Asia (Simões et al., 2023). While fungi threaten human health through invasive infections and antifungal resistance, their impact is equally profound in agriculture, where they devastate staple crops and challenge global food security.

## 7 Fungi and plant health

Fungal pathogens are among the most destructive forces in agriculture, threatening food security and ecosystem stability. The study estimate that fungal diseases account for 15–20% of global crop losses annually, but the impact is magnified by mycotoxin contamination that compromises both yield and safety. They highlight *Fusarium* spp. as a dual threat reducing harvests while producing toxins such as deoxynivalenol and zearalenone that undermine food safety and trigger trade restrictions. This duality illustrates how fungal pathogens simultaneously drive economic losses and nutritional insecurity (Simões et al., 2023).

Recent estimates suggest that fungal diseases, such as rice blast (*Magnaporthe oryzae*) and wheat rusts (*Puccinia* spp.), cause yield losses of 10–30% annually, equating to approximately $70 billion in economic damage and threatening food security for millions (Oliveira-Garcia et al., 2023; Wang et al., 2023) and *Fusarium* head blight representing some of the most damaging examples (Li et al., 2024) often linked to mycotoxin contamination by *Fusarium* species, compromising food safety (Simões et al., 2023). These pathogens employ a sophisticated arsenal of effectors secreted proteins and metabolites that target host immune signaling and metabolic pathways. Advances in molecular plant–microbe interaction studies have revealed that effectors are not randomly distributed but are often encoded near transposable elements, allowing rapid diversification to overcome host defenses (Kainat et al., 2025). Structural studies have further highlighted conserved folds across diverse effectors, despite low sequence similarity, suggesting convergent strategies in manipulating host physiology (Le Naour-Vernet et al., 2025).

Resistance breeding remains a frontline strategy for managing plant diseases, yet resistance genes are frequently overcome by evolving pathogen populations. To address this, genome editing tools are being harnessed to generate more durable forms of resistance. CRISPR/Cas systems have been successfully applied to knock out susceptibility genes, such as MLO in wheat and barley, conferring resistance to powdery mildew without major growth penalties (Chen et al., 2024). Beyond knockout strategies, CRISPR activation (CRISPRa) is emerging as a way to boost expression of defense genes without altering coding sequences, providing a flexible and potentially more durable resistance mechanism (McLaughlin et al., 2025). In legumes and pulse crops, comparative genomic studies are identifying candidate genes for editing, aiming to transfer resistance strategies from model systems to underutilized crops (Singer et al., 2024).

Another critical advance has been understanding effector delivery into host cells. Studies have shown that filamentous pathogen effectors exploit endocytosis to cross plant plasma membranes, highlighting trafficking pathways as key host processes that can be manipulated for resistance (Wang et al., 2023). Direct experimental work further demonstrates that clathrin-mediated endocytosis facilitates the uptake of RXLR-like effectors into host cells, confirming mechanistic details of this process (Oliveira-Garcia et al., 2023). Additionally, unconventional secretion pathways have been reported, bypassing classical signal peptide routes, which may allow pathogens to fine-tune the timing of effector release (Li et al., 2024). These insights are essential for predicting how pathogens adapt and for designing plants with more robust immune surveillance.

Together, the integration of structural biology, effectoromics, and precision genome editing is reshaping the landscape of plant–fungal research. While translation to the field is still limited by regulatory, ecological, and socioeconomic challenges, these tools hold promise for building durable resistance in staple and specialty crops alike. With food security pressures rising under climate change, fungal plant health research is increasingly recognized as a global priority. These advances in understanding plant–fungal interactions set the stage for integrating structural biology, genome editing, and computational tools to address both agricultural and medical challenges.

## 8 Translational frontiers

High-resolution structures are now demystifying some of the hardest fungal targets especially membrane proteins that drive virulence and drug resistance. A striking example is the ABC efflux pump Cdr1 of *Candida albicans*, long implicated in azole resistance but structurally inaccessible until recently (Peng Y. et al., 2024). Cryo-EM resolved multiple Cdr1 states and ligand poses, explaining how azoles are recognized and how the veterinary agent milbemycin oxime locks the pump to block drug efflux a roadmap for rational inhibitor design against clinical resistance (and a template for other ABC transporters). This is the kind of target-level clarity that can seed hit-finding campaigns and medicinal chemistry around bona fide resistance mechanisms (Peng Y. et al., 2024). Beyond experimentally solved structures, AlphaFold 3 now predicts protein–protein, protein–nucleic acid and protein–ligand complexes, extending *in silico* tractability to many fungal assemblies (e.g., effector–host interfaces, multienzyme complexes in ergosterol or cell-wall pathways). Although it’s a server-gated resource, the model’s complex-aware predictions have clear translational value for target assessment, interface mutagenesis, and screening triage (Abramson et al., 2024a,b).

CRISPR pushes from proof-of-concept to routine engineering. Genome editing in filamentous fungi has matured fast. Practical playbooks now cover Cas9/Cas12a choice, donor design, RNP delivery, selection schemes, and multiplex edits across industrial and pathogenic species. Optimized editing workflows now enable scalable pathway engineering, linking high-efficiency CRISPR to translational outcomes like novel metabolite production. Such jumps open the door to turning on silent BGCs, removing competing metabolic sinks, and tuning secretion the nuts and bolts of translating fungal biology into products (enzymes, natural products, vaccines) or attenuated strains for study (Shen et al., 2024). In parallel, CRISPR is pairing with single-cell/spatial readouts to make cause-and-effect tractable in native contexts: pooled editing and barcoded screening in Candida now resolve fitness genes across stress conditions and host-like environments data that flow straight into target nomination and combination-therapy logic (Xiong et al., 2024).

Machine-learning is becoming the connective tissue of fungal pipelines. On the natural products side, models originally trained on bacterial biosynthetic gene clusters (BGCs) have been adapted to fungi to predict secondary-metabolite bioactivity directly from BGC features boosting prioritization of cryptic clusters and cutting wet-lab triage time (Riedling et al., 2024).

At the systems end, spatial metatranscriptomics now maps host tissues and their resident bacteria/fungi simultaneously, resolving micro-scale “hotspots” where plant–microbe–microbe interactions are most active; this is a translational entry point for targeted biocontrol or precision microbiome engineering. In infection biology, single-cell atlases of *Candida auris* skin infection uncover the cell-type–specific immune programs fungi evade actionable signals for host-directed therapy or vaccine adjuvants (Saarenpää et al., 2024). For antifungal resistance (AFR), new resources and models are arriving: curated datasets of resistance mutations (FungAMR, ResFungi) and ML methods to predict AFR biomarkers and targets can feed back into surveillance, diagnostics, and compound design. These tools won’t replace susceptibility testing, but they can pre-score variants, highlight cross-resistance risks, and help labs in low-resource settings triage cases (Bédard et al., 2024; Santana de Carvalho et al., 2024). To help address disparities in access to advanced diagnostic infrastructures, initiatives such as the EU-funded Multi-domain Open MALDI Spectra Archive for Identification of Microorganisms (MALDIbank) aim to provide open, high-quality MALDI-TOF reference spectra, facilitating fungal and microbial identification across laboratories with varying technical resources.

The translational arc is visible in the antifungal pipeline itself. Fosmanogepix (Gwt1 inhibitor) keeps accumulating supportive preclinical and clinical data, with invasive mold disease results reinforcing momentum; olorofim (DHODH inhibitor) faced a Complete Response Letter from the FDA in 2024 but continues toward resubmission and late-stage development illustrating how mechanistic novelty meets regulatory reality (Almajid et al., 2024; Hodges et al., 2025). Meanwhile, structure-informed approaches to efflux inhibition (e.g., Cdr1) and AI-aided lead triage are tightening the loop from target to therapeutic (Ibe and Pohl, 2024; Peng Y. et al., 2024).

Put together, these advances give fungal research the long-missing predict–edit–measure toolkit. With AlphaFold3 and machine learning applied to biosynthetic gene clusters (BGCs), researchers can now generate confident *in silico* hypotheses about protein–protein interactions, effector functions, and secondary metabolite activities (Abramson et al., 2024a; Riedling et al., 2024). At the experimental level, high-efficiency CRISPR workflows across previously intractable filamentous fungi enable causal gene tests and chassis tuning for both pathogenicity studies and industrial applications (Ropero-Pérez et al., 2024; Shen et al., 2024). Complementing this, single-cell and spatial assays provide ground truth in the relevant tissues and microbiomes, capturing the ecological and host contexts in which fungal traits are expressed (Balakumar et al., 2024; Saarenpää et al., 2024). The logical next step is integration: co-designing edits using structure-aware AI, screening engineered strains under spatially realistic conditions, and closing the loop with diagnostics and resistance-aware clinical pipelines. As summarized in Table 1, recent advances across fungal domains highlight actionable paths to mitigate risks and harness opportunities.

## 9 Emerging technologies for rapid identification and characterization of fungi

Accurate and rapid identification of fungi is essential across clinical diagnostics, agriculture, food safety, environmental monitoring, and biodiversity assessment. Traditional morphology-based identification and culture-dependent methods, while foundational, are increasingly complemented or replaced by advanced molecular, computational, and analytical technologies that enable high-resolution fungal detection and characterization in complex samples.

### 9.1 High-throughput sequencing and genomic approaches

Next-generation sequencing (NGS) technologies have transformed fungal identification by enabling culture-independent detection and taxonomic resolution across diverse ecosystems. Amplicon-based sequencing of ribosomal markers, particularly the internal transcribed spacer (ITS) region, remains a cornerstone for fungal community profiling, while long-read sequencing platforms have improved species- and strain-level discrimination by resolving repetitive genomic regions and complex structural variants. Whole-genome sequencing (WGS) further enables comprehensive characterization of fungal pathogens, including virulence determinants, antifungal resistance mechanisms, and population structure, facilitating outbreak tracking and evolutionary analyses in clinical and environmental contexts (Kappel et al., 2024; Xu, 2020).

Metagenomic and metatranscriptomic approaches extend beyond taxonomic identification by capturing functional potential and activity, allowing inference of metabolic pathways, secondary metabolite biosynthesis, and host–fungus interactions directly from environmental or host-associated samples. These approaches are particularly valuable for uncultivable or slow-growing fungi and are increasingly integrated with reference genome databases to improve annotation accuracy (Nilsson et al., 2019; Runnel et al., 2022).

### 9.2 Proteomic and spectrometry-based identification

Mass spectrometry–based technologies have emerged as rapid and cost-effective tools for fungal identification. Matrix-assisted laser desorption/ionization time-of-flight mass spectrometry (MALDI-TOF MS) enables species-level identification within minutes by comparing protein spectral fingerprints to curated databases. While initially developed for bacterial diagnostics, recent expansions of fungal reference libraries have significantly improved its performance for yeasts and filamentous fungi in clinical microbiology laboratories. Beyond identification, proteomic profiling provides insights into stress responses, antifungal resistance, and host-adaptive traits, linking phenotype with molecular function (Motteu et al., 2022; Sa et al., 2025).

### 9.3 Biosensors and isothermal amplification technologies

Rapid point-of-care detection of fungi has advanced through the development of isothermal nucleic acid amplification methods such as loop-mediated isothermal amplification (LAMP) and recombinase polymerase amplification (RPA). These platforms enable sensitive and specific fungal detection without thermal cycling, making them suitable for field deployment in agriculture and low-resource clinical settings. Coupled with lateral flow readouts or fluorescence-based detection, isothermal assays allow near-real-time identification of pathogenic fungi in crops, food products, and clinical samples (Mori and Notomi, 2020; Zhang et al., 2024).

Recent innovations integrate these amplification methods with biosensor technologies, including electrochemical and paper-based sensors, enabling multiplex detection and quantitative analysis. Such systems are increasingly explored for early detection of fungal contamination and disease outbreaks, supporting proactive intervention strategies.

### 9.4 CRISPR-based diagnostics and functional characterization

CRISPR-based detection platforms represent a rapidly evolving frontier in fungal diagnostics. Systems leveraging Cas12 and Cas13 nucleases enable highly specific detection of fungal DNA or RNA through collateral cleavage–mediated signal amplification. These technologies combine the sensitivity of molecular diagnostics with portability and speed, offering promise for on-site fungal detection in clinical, agricultural, and environmental settings. Beyond diagnostics, CRISPR–Cas tools are also widely applied for functional genomics in fungi, enabling targeted gene disruption, transcriptional regulation, and systematic interrogation of virulence and metabolic pathways (Kellner et al., 2019; Li et al., 2023).

### 9.5 Artificial intelligence and integrative computational approaches

Machine learning and artificial intelligence (AI) approaches are increasingly applied to fungal identification and characterization, including image-based species recognition, automated colony morphology analysis, and predictive modeling of antifungal resistance. When integrated with genomic, proteomic, and metabolomic datasets, AI-driven frameworks enable multidimensional profiling of fungal systems, accelerating discovery and improving diagnostic accuracy. Such integrative approaches are particularly valuable for distinguishing closely related species and for predicting pathogenic potential based on molecular signatures rather than taxonomy alone (Mohseni and Ghorbani, 2024).

Collectively, these emerging technologies are reshaping how fungi are detected, identified, and characterized, moving the field toward faster, more precise, and functionally informed diagnostics. Their integration across disciplines is essential for addressing fungal threats to human health, agriculture, and ecosystems while supporting biodiversity assessment and sustainable biotechnology.

## 10 Conclusion

Fungi occupy a paradoxical position at the intersection of ecological resilience, food security, and global health, acting as highly adaptive evolutionary agents whose responses to anthropogenic pressures climate change, agricultural intensification, global trade, and antimicrobial use are reshaping disease landscapes, ecosystem stability, and biotechnological potential across terrestrial and aquatic environments. Their impacts extend well beyond human health to include animal and aquaculture systems, where fungal diseases increasingly threaten biodiversity, food production, and ecosystem services, underscoring the need for genuinely cross-domain perspectives.

Recent technological convergence has transformed fungal research from largely descriptive inquiry into a predictive and translational discipline. The integration of AlphaFold 3–enabled structural modeling, machine-learning-guided biosynthetic gene cluster prioritization, CRISPR-based genome editing and chassis optimization, and single-cell and spatial omics has created a powerful “predict–edit–measure” toolkit. These advances are accelerating antifungal target discovery, enzyme engineering, natural product mining, and the development of sustainable fungal biotechnologies, including mycoproteins, industrial enzymes, and biocatalysts that support circular bioeconomy models spanning agro-food residues, marine byproducts, and pollutant remediation.

At the same time, several interconnected challenges threaten to limit the societal impact of these advances. Accelerating antifungal resistance, exemplified by the global spread of *Candida auris*, remains a critical concern across human and animal health. Large gaps persist in fungal biodiversity knowledge, particularly in undersampled regions and aquatic systems, constraining risk assessment and bioprospecting efforts. In parallel, translational bottlenecks including limited access to advanced diagnostics, regulatory constraints on environmental deployment of genome-edited organisms, and inequities in research infrastructure continue to impede the transfer of laboratory innovations to real-world applications.

Addressing these challenges requires coordinated action across the One Health continuum. Key priorities include integrated surveillance frameworks that encompass human, animal, plant, and environmental fungal threats; expanded access to diagnostic and data-sharing infrastructures; translational pipelines that combine biological, chemical, and computational strategies; sustainability-by-design principles in fungal biotechnology; and cross-sector collaboration that bridges medical, agricultural, environmental, and industrial mycology. The coming decade represents a critical window in which climate change, antimicrobial resistance, and biodiversity loss are accelerating alongside unprecedented technological capacity. Harnessing fungal biology as a resource for planetary health will depend on recognizing fungi not as isolated threats or tools, but as dynamic partners whose evolutionary versatility can be directed toward resilient, sustainable solutions through coordinated and inclusive global action.
